# Comparative analysis of microbial composition and functional characteristics in dental plaque and saliva of oral cancer patients

**DOI:** 10.1186/s12903-024-04181-1

**Published:** 2024-04-04

**Authors:** Man Zhang, Yiming Zhao, Abdulrahim Umar, Hailin Zhang, Lirong Yang, Jing Huang, Ying Long, Zheng Yu

**Affiliations:** 1 Translational Medicine Center, Department of Head and Neck Surgery, Hunan Cancer Hospital, The Affiliated Cancer Hospital of Xiangya School of Medicine, Central South University, Changsha, Hunan China; 2 Human Microbiome and Health Group, Department of Microbiology, School of Basic Medical Science, Central South University, Changsha, Hunan China; 3 Department of Parasitology, School of Basic Medical Science, Central South University, Changsha, Hunan China

**Keywords:** Oral cancer, Dental plaque, Saliva, Oral microbiota, Herpes simplex virus 1

## Abstract

**Background:**

The oral cavity is home to various ecological niches, each with its own unique microbial composition. Understanding the microbial communities and gene composition in different ecological niches within the oral cavity of oral cancer (OC) patients is crucial for determining how these microbial populations contribute to disease progression.

**Methods:**

In this study, saliva and dental plaque samples were collected from patients with OC. Metagenomic sequencing was employed to analyze the microbial community classification and functional composition of the different sample groups.

**Results:**

The results of the study revealed significant differences in both the function and classification of microbial communities between saliva and dental plaque samples. The diversity of microbial species in saliva was found to be higher compared to  that in plaque samples. Notably, *Actinobacteria* were enriched in the dental plaque of OC patients. Furthermore, the study identified several inter-group differential marker species, including *Prevotella intermedia*, *Haemophilus parahaemolyticus*, *Actinomyces radius*, *Corynebacterium matruchitii*, and *Veillonella atypica*. Additionally, 1,353 differential genes were annotated into 23 functional pathways. Interestingly, a significant correlation was observed between differentially labeled species and Herpes simplex virus 1 (HSV-1) infection, which may be related to the occurrence and development of cancer.

**Conclusions:**

Significant differences in the microbial and genetic composition of saliva and dental plaque samples were observed in OC patients. Furthermore, pathogenic bacteria associated with oral diseases were predominantly enriched in saliva. The identification of inter-group differential biomarkers and pathways provide insights into the relationship between oral microbiota and the occurrence and development of OC.

**Supplementary Information:**

The online version contains supplementary material available at 10.1186/s12903-024-04181-1.

## Introduction

The oral cavity serves as the initial stage of the human digestive system, where daily feeding actions gradually establish a diverse ecosystem of up to 1,000 total microbial species that can have either positive, negative, or digestive-supporting effects on humans [1]. Over time, researchers have reported *Streptococcus* species as dominant in a diverse and balanced community of key bacterial species found in a healthy oral microbiota, which include *Lactobacillus*, *Actinomyces*, *Veillonella*, *Haemophilus,* and *Neisseria* that makes up to 85.4% of all adults’ genera and 71.1% of youth genera in their respective samples [2]. This diverse ecosystem of oral microbiota plays a crucial role through functional pathways in maintaining oral health, preventing dental diseases, promoting proper digestion and nutrient absorption [3]. Although, factors such as poor oral hygiene practice, unhealthy diet and genetic disorders can result to development of oral disease [4].

OC is the predominant type among the Head and Neck cancer, ranking as the sixteenth most common cancer globally [5, 6]. Research has shown a close association between the oral microbiota and tumor development [7]. This oral microbiota normally resides in both saliva and surface areas of the oral cavity as planktonic bacteria and dental plaque respectively [8]. Dental plaque is a structured microbial biofilm, and the organic acids produced by the sugar metabolism of dental plaque biofilm play a crucial role in lowering the pH of the tooth surface and demineralization [9]. Poor dietary habits such as frequent consumption of sugar can induce dysbiosis of the microbial community on the gum, leading to the development of dental caries [10]. The oral diseases caused by dysbiosis in the oral microbiota (such as periodontitis) are associated with various systemic disorders [11]. Oral bacteria and their metabolic products enter the bloodstream, increasing oral and systemic inflammation through the release of toxins or microbial by-products [12, 13]. Much research has reported the associated risk of periodontal disease and systemic diseases such as cardiovascular disease, diabetes, respiratory infections, and adverse pregnancy outcomes, with a 19% increased risk in cardiovascular diseases and an elevated risk of gaining the critical COVID-19 infection [14, 15]. AlsoMoreover, the dysbiosis of the oral microbiota can be used to potentially predict the risk of cancer, and colonization by specific microbiota might be important for the development of OC and related diseases [16]. The host’s immune response to certain oral bacteria and their byproducts can trigger chronic inflammation, which subsequently results in development and progression of oral cancer [17].

Exploring the microbial changes that occur during oral carcinogenesis is crucial in identifying diagnostic and prognostic biomarkers for early detection and monitoring of oral cancer [18]. Investigating oral microbiota functional pathways in oral cancer patients can reveal disease mechanisms and intricate interactions between microbial species, host cells, and the immune system [19]. Furthermore, conducting longitudinal studies would provide researchers with the opportunity to assess dynamic changes that may occur over time and potentially identify precise therapeutic agents for the treatment of OC. With the advent of metagenomic sequencing technology and a deeper understanding of bacterial communities, the relationship between the dysbiosis of the human microbiota and the pathogenesis of various diseases is gradually becoming clearer [20]. However, the oral cavity harbors numerous ecological niches, each with its own unique microbial community, with a lack of exploration into the utilization of metagenomic sequencing technology to analyze the oral microbiota [21]. The characteristics and differences in the composition and functional features of the saliva and dental plaque microbiomes in oral cancer patients remain unknown. Additionally, there remains a limited understanding of the comprehensive mechanisms underlying the development of oral cancer, and it continues globally to have a high incidence rate and low survival rate among its patients [22].

In this study, we compared the differences in the oral microbiota and genomic composition of saliva and dental plaque between OC patients, identifying a set of potential differential markers and genes. The relationship between microorganisms and functional pathways was revealed through functional enrichment and correlation analysis, which was expected to provide further understanding of the relationship between microorganisms and the occurrence and development of OC. This comparative study could further improve our understanding of the pathogenesis of OC and propose potential therapeutic targets for treating or improving the quality of life for patients with OC.

## Materials and methods

### Subject recruitment and sample collection

To investigate the microbial and functional genetic features in OC patients, a total of 5 saliva samples and 5 dental plaque samples were collected from patients with OC at Hunan Cancer Hospital. All participants providing samples informed consent and had complete clinical and pathological data. To minimize potential confounding factors, the inclusion criteria for participants were as follows: (1) No other malignant tumors were detected during systemic examination, excluding distant metastasis. (2) Avoidance of smoking, alcohol consumption, and eating for at least 30 minutes prior to sample collection. (3) No immunosuppressive medication within the past 6 months. (4) No history of severe periodontal disease, severe dental caries, or oral mucosal diseases in the past 3 months; no other systemic diseases; no history of oral surgery; and no history of antibiotic use. (5) Patients without a history of oral infectious diseases or bleeding. In accordance with the Helsinki Declaration, the cases included in this study were collected and approved by the ethics committee of Hunan Cancer Hospital (Ethics Approval Number: KYJJ-2023-025). Patients were informed about the sample collection procedures and signed informed consent forms.

Before collecting the samples, the participants were instructed to rinse their mouths with distilled water to remove any residual food debris. The swab was gently rubbed back and forth three times on the upper and lower incisors, first molars, and first bicuspid. It was then placed in a sterile collection tube. 1 ml of sterile PBS buffer was added to a sterile collection tube, ensuring that the swab was fully immersed in the elution buffer to allow for complete dissolution of the sample. Subsequently, the sample tube was centrifuged at 12,000 rpm for 10 minutes at 21 ℃ (Glanlab, Changsha, China). This step was repeated three times to collect the eluted buffer. Saliva was collected in a sterile tube with a minimum volume of 1 ml.

### DNA extraction and metagenomic sequencing

All samples were accessed for metagenomic sequencing. Total microbial genomic DNA was extracted using the QIAGEN DNeasy Power Water Kit (14900-100-NF). The microbial DNA that was extracted underwent processing to create metagenomic sequencing libraries with 400 bp insert sizes, utilizing the Illumina TruSeq Nano DNA LT Library Preparation Kit. Subsequently, each library underwent sequencing on the Illumina HiSeq X-ten platform (Illumina, USA), employing the PE150 strategy. Potential 3' end adapter sequences were identified, and the sequence was truncated at the identified adapter sequence (R1: AGATCGGAAGAGCACACGTCTGAACTCCAGTCA; R2: AGATCGGAAGAGCGTCGTGTAGGGAAAGAGTGT).

### Data processing

The raw sequencing data contained various issues, such as short sequences, excessive ambiguous bases, and contamination from adapters. To ensure data accuracy, we performed further quality control on the raw sequences, primarily to remove contamination and human host sequences. Initially, we utilized Fastp (version 0.23.2) to eliminate low-quality reads, specifically those with an average quality score < 15 and a length less than 15 bases [23]. FastUniq (version 1.1.0) was utilized as a tool to remove duplicate sequences, processing PCR duplicates, or redundant sequences caused by PCR amplification in DNA sequencing data [24]. We employed Bowtie2 (version 2.5.1) to align the sequences against the human genome database (hg38) and filter out sequences of human origin [25]. Initially, species annotation on the gene sequences was performed using Kraken2 (version 2.1.2), resulting in taxids (unique identifiers for NCBI taxonomic units) being obtained for every gene [26]. Following that, the names and taxonomy of these taxids were translated into corresponding species information, ranging from phylum to species. Then, the high-quality reads were reconstructed from the quality-controlled data using MEGAHIT (version 1.2.9) [27]. Following the assembly, gene prediction was performed on the MEGAHIT assembly contigs using Prodigal (version 2.6.3) [28]. EggNOG-mapper (version 2.0.1) was used to perform the function annotation for the gene catalog [29]. Subsequently, clustering and redundancy removal of the gene sequences were carried out using CD-HIT (version 4.7) with a 90% global sequence similarity threshold [30]. The relative abundance of differential genes was then estimated using salmon (version 0.13.1) [31].

### Statistical analysis and visualization

The data analysis and visualization in this study were performed using R (v.4.2.3). Based on the Spearman correlation matrix, two co-occurrence networks were constructed. The False Discovery Rate (FDR) correction by the Benjamini-Hochberg (BH) method was applied to correct for multiple hypothesis testing. The Spearman correlation coefficient was found to be 0.8, and the adjusted *P*-value was 0.05. Subsequently, the co-occurrence networks were visualized using Gephi software (v.0.9.5) [32]. The visualization of microbial community composition was conducted using the ggbarplot function from the ggpubr package (v.0.6.0). The Wilcoxon test was utilized to compare the relative abundance differences of different species between groups, and the ggboxplot was utilized for visualization. A *p*-value less than 0.05 was considered statistically significant. Alpha diversity was calculated based on species abundance using the diversity function from the vegan package (v.2.6-4) [33]. To describe the degree of similarity among various microbial communities within various groups, beta diversity was employed. In this work, the diversity index of the microbiota in saliva and dental plaque samples was determined using the “vegdist” function, which is based on the Bray-Curtis distance. The sample groups were subjected to PCoA analysis using the PERMANOVA method, with 999 permutations used for statistical significance testing. ANOSIM was used to test the significance of differences between two groups [34]. Non-metric multidimensional scaling (NMDS) was conducted using the metaMDS function from the vegan package for ordination analysis [35]. The volcano plot was generated using the DEseq2 package, with labeling of species and genes that met the criteria of |log_2_FoldChange| > 2 and *P* < 0.05 [36]. To show the link between genes in various samples, the Pearson correlation coefficient was employed. Better biological repeatability was suggested by a correlation coefficient near 1, which showed a closer biological link between the genes in the samples [37]. Similarly, the Pearman correlation coefficient was also employed to assess the correlation between different microbial species and genes [38]. A T-test was employed to calculate the abundance differences in functional pathways between groups.

## Result

### Characteristics of microbial community composition in saliva and dental plaque

**Fig. 1:**
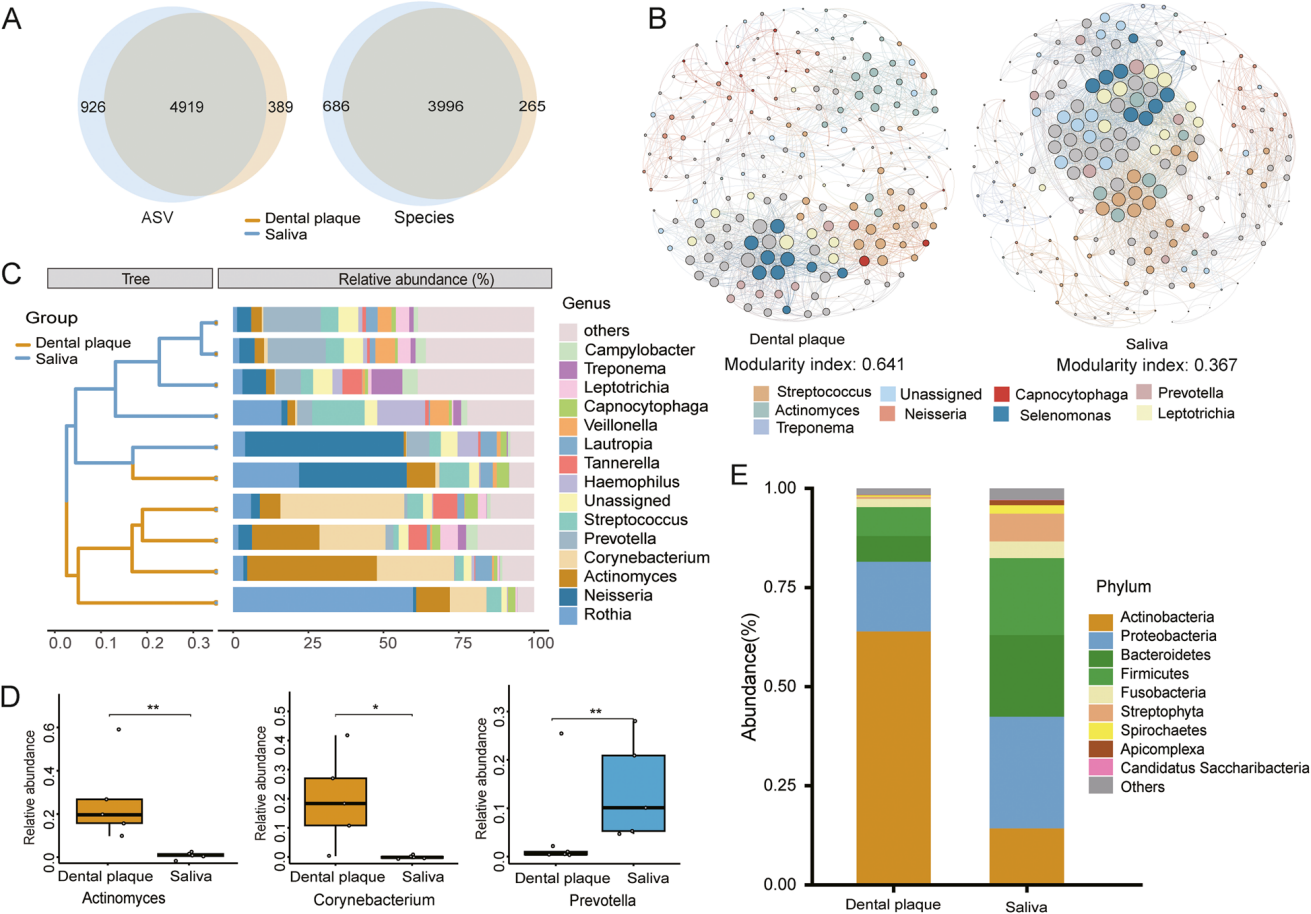
Composition of microbiota in saliva and dental plaque samples. (**A**) The Venn plot shows the overlap and differences between the microbiota of dental plaque and salivaa. The blue area represents saliva samples, and the yellow area represents dental plaque samples. (**B**) Microbial network composition of dental plaque and saliva, with different colored points denoting different species and each species' contribution indicated by the size of the point. (**C**) Relative abundance of species composition at the genus level in the two groups, with lines connecting samples from saliva (blue) and dental plaque (yellow). (**D**) Wilcoxon test, indicating significance levels for *Actinomyces*, *Corynebacterium*, and *Prevotella* (* *P* < 0.05, ** *P* < 0.01). **E** Relative abundance of species composition at the phylum level

To investigate the microbial composition of saliva and dental plaque, a total of 6,234 ASVs (Amplicon Sequence Variant) were extracted from all the samples. Dental plaque and saliva shared 4,919 ASVs, while dental plaque had 328 unique ASVs and saliva had 926 unique ASVs. At the species level, a total of 4,947 species were detected, of which 3,996 species were shared by both sample groups (Fig. 1A). A network diagram was used to visualize the overall community characteristics of dental plaque and saliva microbiota (Fig. 1B). There were differences in the microbial network structure between dental plaque and saliva. The modularization coefficient, which reflects the degree of modularity in the microbial network, was higher in dental plaque (0.641) compared to saliva (0.367). At the phylum level, *Actinobacteria* was significantly enriched in dental plaque (*P* < 0.01) (Supplementary Figure S1), whereas *Proteobacteria*, *Firmicutes*, and *Bacteroidetes* showed a higher relative abundance in saliva compared to dental plaque (Fig. 1E). At the genus level, *Rothia*, *Actinomyces*, and *Corynebacterium* were predominantly enriched in dental plaque, while *Prevotella*, *Streptococcus*, *Neisseria,* and *Veillonella* exhibited a higher relative abundance in saliva (Fig. 1C, D). Additionally, a greater diversity of low-abundance bacteria (other bacteria) was found in saliva, and we also observed a higher relative abundance of *Porphyromonas gingivalis* in saliva (Supplementary Figure S2).

### Analysis of microbial community diversity and species differences

**Fig. 2:**
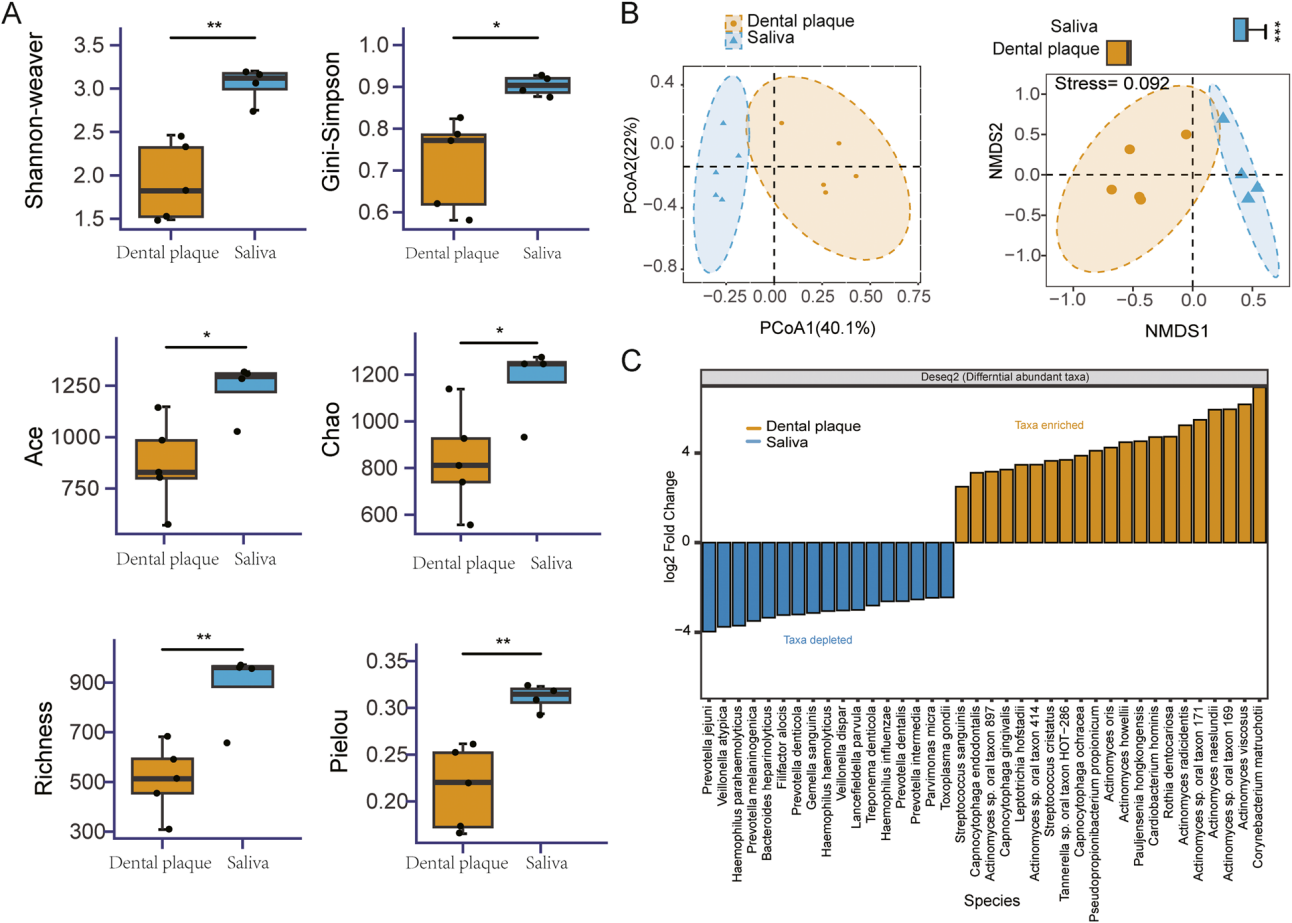
The microbial diversity and differential abundance analysis of microbial communities. (**A**) Alpha diversity was described using Shannon-weaver, Gini-Simpson, ACE, Chao, Richness, Pielou indices. (**B**) Beta diversity was presented by PCoA (ANOSIM, *R* = 0.748, *P* = 0.012) and NMDS analysis (Stress = 0.092). (**C**) Significant analysis of differential species by DEseq2 at the genus level (|log_2_ Fold Change| > 2, *P* < 0.05)

The Shannon-Weaver, Gini-Simpson, Chao1, ACE, Richness, and Pieou indices were calculated at the species level (Fig. 2A). The results showed the six indices for saliva microbiota were significantly higher than those for dental plaque. It demonstrated that the species richness and diversity of microbial communities in the saliva of OC patients were significantly higher than those in dental plaque. The PCoA analysis indicated that the microbial community composition of the two groups was different (Fig. 2B). The ANOSIM test (*R* = 0.748, *P* = 0.012) further demonstrated that the inter-group differences in microbial composition were significantly greater than the intra-group differences (Supplementary Figure S3). The composition of the microbial community was effectively simulated by the NMDS analysis (Supplementary Figure S4). The NMDS analysis result revealed that samples in the two groups had different community compositions (Stress = 0.092) (Fig. 2B). Then, a batch of differential marker species was chosen from the top 100 species based on their relative abundance (*P* < 0.05, |log_2_FoldChange| > 2). *Prevotella spp*., *Haemophilus spp.*, *Actinomyces spp*., *Corynebacterium matruchotii*, and *Veillonella atypica* were identified as differential species between the two groups. *Prevotella spp.* and *Haemophilus spp.* were significantly enriched in saliva, while *Actinomyces spp.* and *Corynebacterium matruchotii* were significantly enriched in dental plaque (Fig. 2C).

### Screening of differential gene and correlation analysis between species and functional pathways

**Fig. 3:**
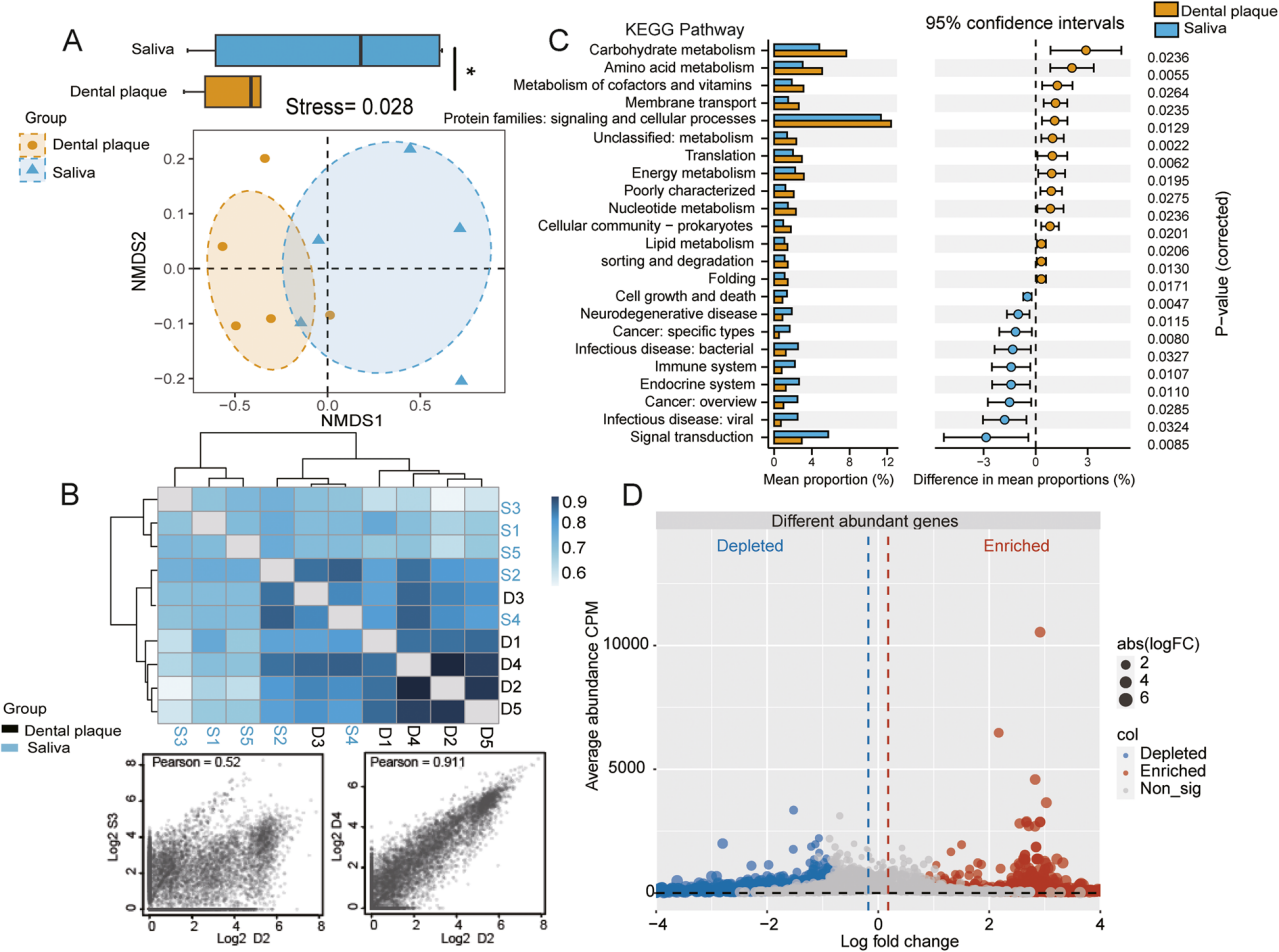
Gene composition and functional annotation of saliva and dental plaque in cancer patients. (**A**) PCoA analysis (80% confidence interval). (**B**) Correlation test between samples at the genetic composition level. The distance between points in a scatter plot represents the correlation between two genes. (**C**) Analysis of inter group differences in functional pathways with the top 50 abundances at level 2 of the KEGG database (T-test, *P* < 0.05). The color of the bars represents different groups, and the length of the bars represents abundance information. (**D**) Volcano plot showing the relative abundance differences of genes, where red points indicate significantly higher relative abundance of the gene in the saliva group (*P* < 0.05, log_2_FoldChange > 2). Blue points indicate significantly lower relative abundance in the saliva group (*P* < 0.05, log_2_FoldChange < -2), and grey points indicate no significant difference

From the perspective of gene composition, the gene results from metagenomic sequencing were transformed into a two-dimensional coordinate system using NMDS analysis. The genomic composition of the two groups differed significantly, as seen by the distinct confidence ellipses of the saliva and dental plaque samples (*P* < 0.05) (Fig. 3A). To further explore the similarity of samples, the Pearson correlation coefficients for gene correlations between different samples were calculated. It was evident from the heatmap that samples within the same group exhibit higher Pearson coefficients, indicating greater genetic similarity within the sample groups and closer microbial relatedness (Fig. 3B). Through T-test analysis, a total of 23 functional gene pathways with significant differences were identified in both saliva and dental plaque samples, including Carbohydrate metabolism, Amino acid metabolism, and Metabolism of cofactors and vitamins (*P* < 0.05) (Fig. 3C). The relative abundance of 1,353 genes showed significant differences between saliva and dental plaque samples (*P* < 0.05, |log_2_FoldChange| > 2). Using dental plaque samples as the control, 735 genes were significantly elevated in saliva samples, whereas 618 were significantly downregulated (Fig. 3D).

**Fig. 4:**
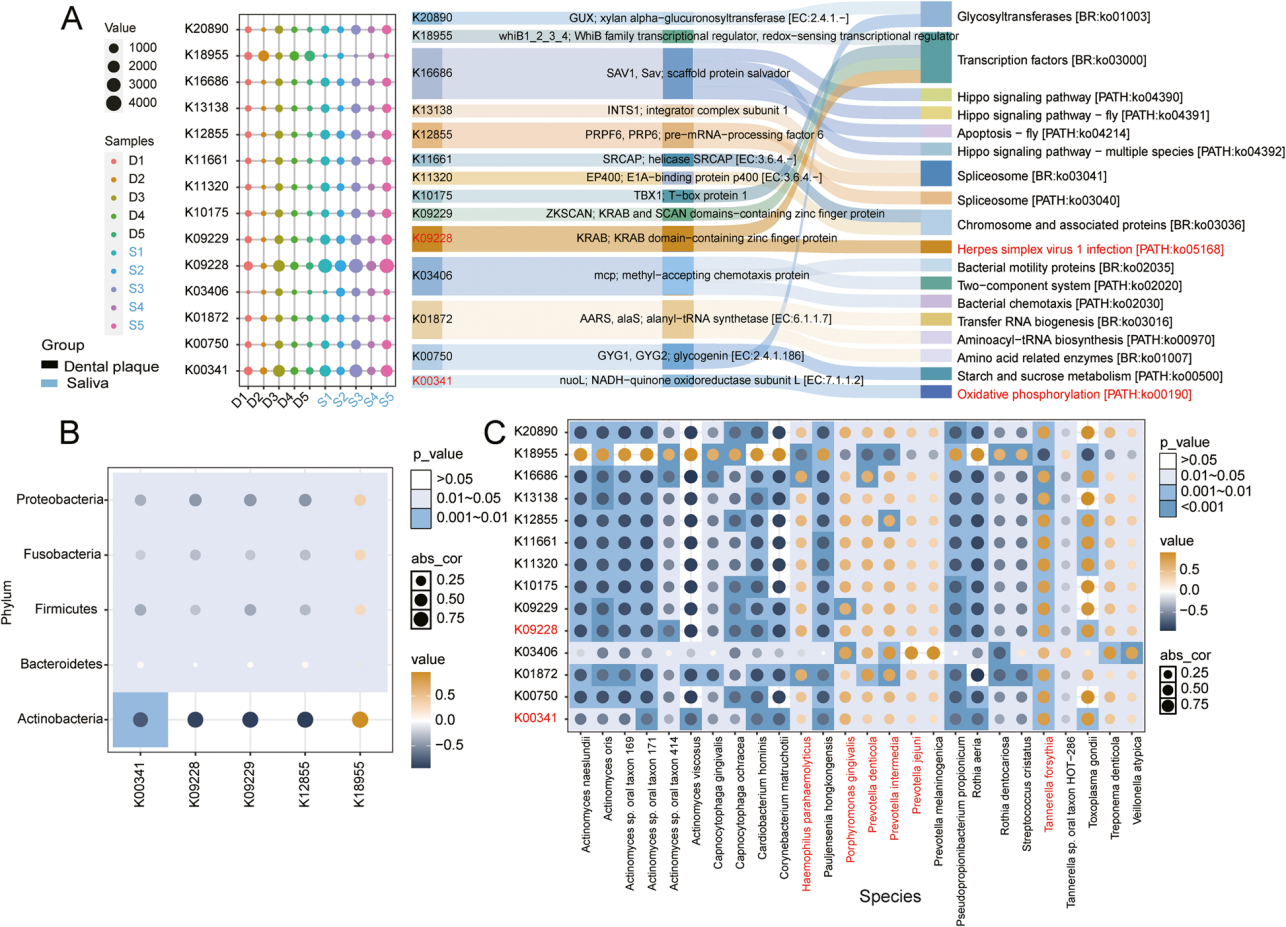
Correlation analysis between differential genes and species. (**A**) Bubble plot (left): composition of the abundance of the top 14 differential genes among different groups. Rows represent differential genes, and each column represents a different sample. Sankey diagram (right): proteins associated with the top 14 differential genes and their corresponding functional pathways. (**B**) Correlation analysis between the top 5 differential genes and the top 5 species at the phylum level based on relative abundance. (**C**) Correlation analysis between the top 14 differential genes and the top 26 differentially abundant species. The size of the circular dots is used to indicate the magnitude of the correlation coefficient, while the color represents the sign of the correlation coefficient. A separate square color block is added to indicate the *p*-value of the correlation test

Furthermore, based on the gene abundance ranking, we identified a total of 14 differential genes among the top 50 ranked genes in abundance. A bubble plot was generated to illustrate the abundance levels of the genes across different samples, with K18955 enriched in dental plaque samples and the remaining 13 genes enriched in saliva samples (Fig. 4A). According to functional annotation, the K18955 gene enriched in dental plaque was involved in the pathway of transcription factors [BR: ko03000], which involved multiple transcription factors participating in the process of gene transcriptional regulation. Among the top ten genes in abundance, five genes (K09228, K00341, K09229, K12855, and K18955) showed significant differences in abundance between the two groups. Especially, K09228 and K00341 were genes with the highest relative abundance across all samples. Based on functional annotations, the K09228 gene was primarily involved in the functional pathways of transcription factors [BR: ko03000] and Herpes simplex virus 1 infection [PATH: ko05168]. The K00341 gene was mainly associated with the pathway of oxidative phosphorylation [PATH: ko00190]. Next, Pearson correlation analysis was performed between the top 5 differentially abundant genes and the top 5 phylum-level species based on relative abundance ranking (Fig. 4B). A strong inverse relationship (*P* < 0.01) between *Actinobacteria* and oxidative phosphorylation [PATH: ko00190] was revealed. This implied that these two factors could be in mutual competition or inhibited under certain circumstances, suggesting that *Actinobacteria* growth and survival may be impacted by oxidative phosphorylation-related metabolic processes. Moreover, based on the relative abundance ranking, the top 50 genes and species with significant differences in relative abundance between groups were selected for correlation analysis (*P* < 0.05, |log_2_ Fold Change| > 2) (Fig. 4C). Oxidative phosphorylation exhibited a significant negative correlation with species mainly belonging to *Actinomyces*, and a significant positive correlation with species such as *Haemophilus parahaemolyticus*, *Tannerella forsythia*, *Porphyromonas gingivalis,* and *Prevotella intermedia*. A significant positive correlation between *Haemophilus parahaemolyticus*, *Prevotella denticola*, *Prevotella intermedia*, *Porphyromonas gingivalis*, *Tannerella forsythia*, and HSV-1 infection was also observed. Furthermore, *Prevotella intermedia* mostly affected genes K01872, K03406, K12855, and K18955, whereas *Porphyromonas gingivalis* considerably affected the pathways ko02035, ko02020, ko02030, and ko03000.

## Discussion

The study discovered that the genomic composition, microbial community composition, alpha diversity, beta diversity, and ecological networks of saliva and dental plaque in OC patients were altered. Genes and microorganisms that showed differences were chosen for correlation analysis. Saliva had a greater variety of microorganisms than dental plaque, including a greater number of low-abundance microorganisms and a microbial network with less modularity. Differential marker candidates were found. The genomic composition was screened for 1,353 differential genes, and the relationship between differential microorganisms and genes was investigated. Most microorganisms and functional pathways exhibited significant correlations.

Our study revealed significant differences in the microbial composition of saliva and dental plaque between cancer patients. The site-specific hypothesis proposed that the microbial communities in different oral sites vary in composition and proportion, and the microbial composition also varies among different ecological niches [39]. The microbes in the saliva were dominated by the *Streptococcus*, *Prevotella*, and *Veillonella* *genera*, which comprise 70% of this microbiota [8]. Their transient and free-floating nature classifies them as planktonic bacteria with a unique ability to interact with and influence various factors, such as oral hygiene. Although, attached bacteria species such as *Streptococcus mutans*, *Porphyromonas gingivalis*, *Prevotella intermedia*, *Fusobacterium nucleatum*, and *Actinomyces* formed the dental plaque through a process of pellicle formation, bacterial adhesion, colonization, matrix formation, and maturation, forming dental plaque to develop synergistic or antagonistic relationships that could influence oral health or contribute to the development of oral diseases [2].

Based on our results, the relative abundance of *Prevotella*, *Porphyromonas gingivalis,* and *Streptococcus* was also found to be more enriched in saliva. Previous studies have shown that *Prevotella* is frequently present in oral tumor tissues [40, 41]. *Prevotella intermedia* and *Porphyromonas gingivalis* were associated with the occurrence and development of cancer [42]. According to reports, it was because the proteases they secrete can degrade host tissues such as the extracellular matrix (ECM), modulate host immune responses, and participate in cell proliferation and apoptosis [43, 44]. Pathogenic bacteria lik*e Porphyromonas gingivalis* and *Fusobacterium nucleatum* in the oral cavity can upregulate inflammatory mediators and facilitate invasion and spreading to adjacent cells, promoting chronic inflammation. This chronic inflammation can lead to changes in cell metabolism, proliferation, and the development of tumors [45]. Genetically, *Lactobacilli* and *Streptococci* species produce reactive oxygen species, nitrogen reactive species, sulfides, nitrosamines, and acetaldehydes that can lead to DNA damage in epithelial cells and thus promote tumorigenesis [46, 47]. Hypomethylation of interleukin-6 and interleukin-8 gene promoters has been linked to overexpression of these cytokines in inflamed periodontal disease tissues compared to control tissues. Consequently, the release of proinflammatory mediators can stimulate human gingival epithelial cells, leading to the recruitment and activation of inflammatory cells, potentially facilitating oral malignant transformation [48].

Additionally, the diversity and abundance of species in saliva exceeded those in dental plaque. On one hand, the fluid nature of saliva allows it to collect and carry microbial communities from different oral sites, such as the tongue coating and buccal mucosa. It can lead to a higher diversity of microorganisms in saliva, as it may be influenced by other parts of the oral cavity. However, it is important to note that many microorganisms in saliva do not exhibit high abundance [49]. On the other hand, due to the lubricating, protective, and digestive functions of saliva within the oral environment, various microorganisms may have a broader distribution and survival in the oral cavity through saliva. In contrast, dental plaque is a biofilm that adheres to the tooth surface, and its relatively enclosed environment may result in a relatively lower diversity and abundance of microorganisms [50]. A microbial comparative study of periodontitis and a healthy group in terms of alpha diversity reported much more substantial differences in bacterial diversity than the supra-gingival plaque in a saliva sample [51]. The more diverse and abundant microbial community in the saliva are implicated in periodontal diseases with *Porphyromonas gingivalis*, *Tannerella forsythia,* and *Fusobacterium alocis* as causative agents. These causative agents have proven to be moderately accurate at distinguishing the severity of periodontal disease in a full mouth probe [52].

In our research findings, the saliva microbiota of OC patients exhibited a dysbiotic state, characterized by an increased abundance of *Neisseria*, *Prevotella*, *Haemophilus*, *Corynebacterium*, *Streptococcus*, *Capnocytophaga*, and *Porphyromonas*. Numerous studies have indicated a close correlation between the increased abundance of these bacteria and the occurrence and progression of cancer, but the specific mechanisms underlying this relationship require further investigation [53, 54]. Another study of the salivary microbiota reported Streptococcus dominance with approximately 25 and 50% of all DNA and RNA reads respectively. Also, a significant disease-associated higher relative abundance of periodontal pathogens such as *Porphyromonas gingivalis* and *Filifactor alocis* was found using both metagenomic and metatranscriptomic analysis [55]. Further comparison of the microbiota in dental plaque and saliva of OC patients revealed that *Actinomyces* was highly enriched in dental plaque. A previous study indicated that *Actinomyces*, as a key microorganism, contributes to the formation of oral biofilms, dental plaque, halitosis, and dental caries by inducing acid and gas production on the tooth surface [56]. However, current studies on the pathogenesis of OC are still very limited, and the relationship between microorganisms and cancer occurrence and development cannot be ignored [22].

Various factors, such as bacteria, viruses, diet, radiation, and genetic mutations, can all contribute to the development of OTSCC [57]. In our study, significant positive correlations were observed between bacteria such as *Porphyromonas gingivalis*, *Haemophilus parahaemolyticus*, *Prevotella intermedia*, and HSV-1 infection. The presence of these bacteria alongside HSV-1 infection may indicate a microbial community that could collectively influence the oral environment and potentially contribute to the development or progression of OC. Further research is needed to explore the mechanisms underlying these correlations and their implications for oral health management and disease prevention strategies. Previous studies have linked oral tongue squamous cell carcinoma (OTSCC) to HSV-1, which may interfere with the cell cycle, activate cell proliferation pathways, and inhibit immune responses, potentially contributing to OC [58]. Furthermore, HSV-1 infection may increase OC risk through interaction with other carcinogenic factors. HSV-1 is believed to act in conjunction with other carcinogens, enhancing tumorigenic effects in the development of oral squamous cell carcinoma (OSCC) [59]. Previous studies detected HSV DNA in 15% to 56% of oral cancer patients [60, 61]. These findings underscore the potential role of HSV-1 in the development of oral cancer and highlight the importance of further research in this area.

This small-scale study aimed to elucidate the differences in saliva and dental plaque microbiota and genomic composition in OC patients. However, the study has certain limitations. Firstly, the sample collection was limited to saliva and dental plaque samples from OC patients, with a relatively small sample size and a lack of data from a control group of healthy individuals. Secondly, while the study observed a significant correlation between oral microbiota and HSV-1 infection, the specific mechanisms underlying this correlation remain unclear. Lastly, the precise role of the microbiota in the pathogenesis of OC is still not well understood.

## Conclusion and future perspectives

Our study found that OC patients exhibit significant differences in microbial composition, gene composition, and function between saliva and dental plaque. OC patients had higher diversity and richness of saliva microbiota, as well as a higher relative abundance of bacteria associated with oral diseases. We also analyzed the close relationship between microorganisms and functional pathways, which may be related to the development of OC. In future, we need to further explore the mechanism of microbial impact on OC through a combination of multidisciplinary and multi omics approaches. This is essential for the early detection and monitoring of diagnostic and prognostic biomarkers, as well as understanding disease mechanisms and complex interactions between microorganisms, host cells, and the immune system.

### Supplementary Information

**Supplementary Material 1.**

## Data Availability

The raw sequence data reported in this paper have been deposited in the Genome Sequence Archive [62] in National Genomics Data Center [63], China National Center for Bioinformation / Beijing Institute of Genomics, Chinese Academy of Sciences (GSA: CRA013876) that are publicly accessible at https://ngdc.cncb.ac.cn/gsa.

## Abbreviations

OC: Oral cancer
ASV: Amplicon Sequence Variant
PCoA: Principal co-ordinates analysis
ANOSIM: Analysis of similarities
NMDS: Non-metric multidimensional scaling
PERMANOVA: Permutational multivariate analysis of variance
HSV-1: Herpes simplex virus 1
ECM: Extracellular matrix
OTSCC: Oral tongue squamous cell carcinoma
OSCC: Oral squamous cell carcinoma
