# Supplementary material for: Comparative analysis of microbial composition and functional characteristics in dental plaque and saliva of oral cancer patients
Source: BMC Oral Health. 2024 Apr 4;24:411. doi: 10.1186/s12903-024-04181-1 (PMC10993480; doi:10.1186/s12903-024-04181-1)
Supplement: Supplementary file 1 — Supplementary Material 1. [file 12903_2024_4181_MOESM1_ESM.docx]

Supplementary Material

Comparative Analysis of Microbial Composition and Functional Characteristics in Dental Plaque and Saliva of Oral Cancer Patients

**Man Zhang^1,2^, Yiming Zhao^2^, Abdulrahim Umar^2^, Hailin Zhang^1^, Lirong Yang^1^, Jing Huang^3^, Ying Long^1^*, Zheng Yu^2^***

*** Correspondence:**

Zheng Yu: [yuzheng@csu.edu.cn](mailto:yuzheng@csu.edu.cn)

Ying Long: longying@hnca.org.cn

# Supplementary Figures


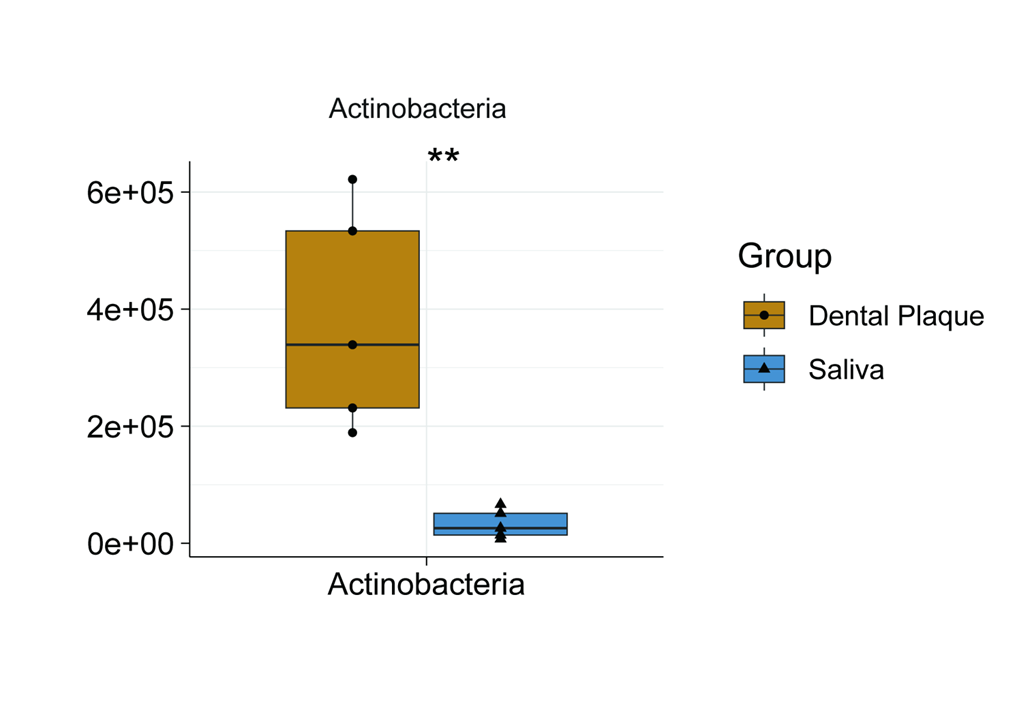


**Supplementary Figure S1. Nonparametric testing of species abundance at the phylum level.** The abundance difference test of *Actinobacteria* in dental plaque and saliva.


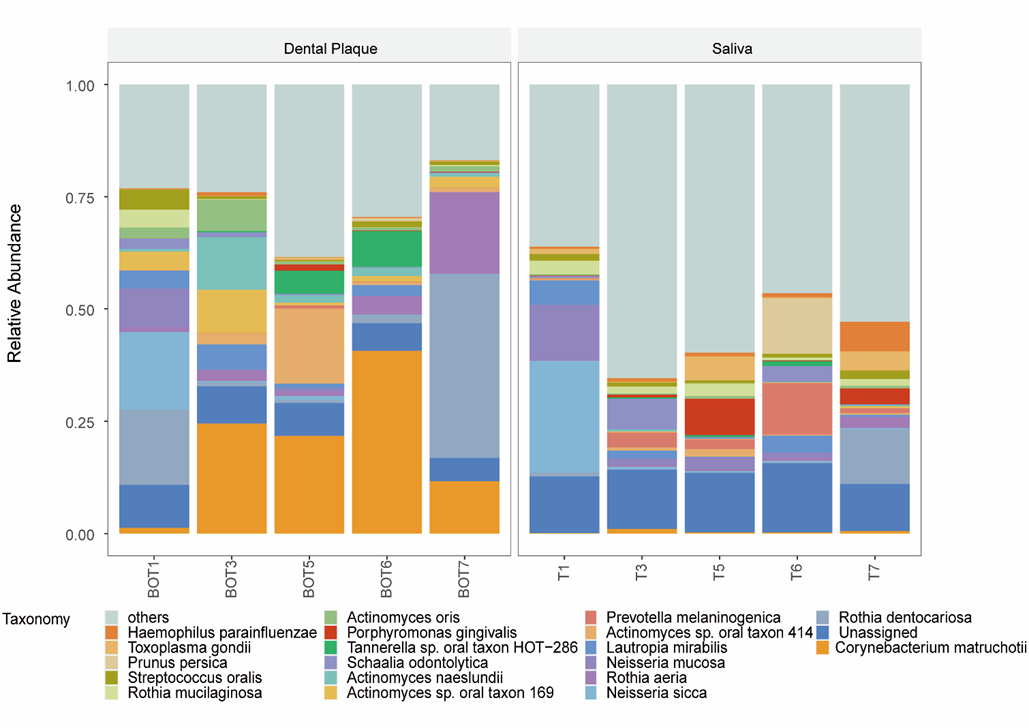


**Supplementary Figure S2. Species level microbial community composition.** Relative abundance of species composition at the species level in the two groups.

**
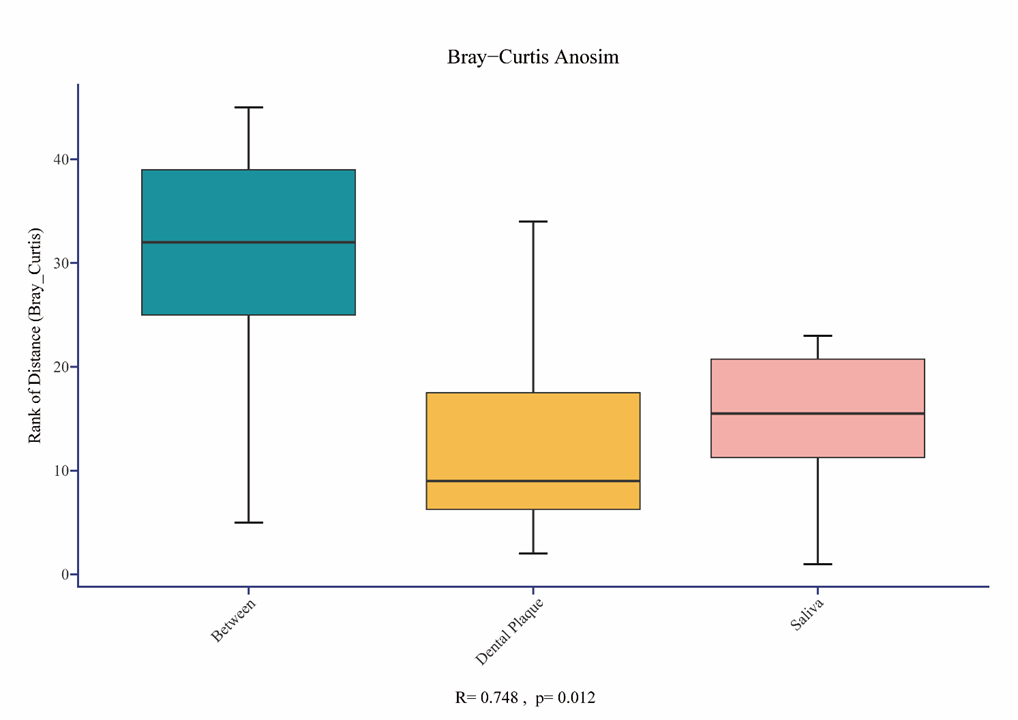
**

**Supplementary Figure S3.** **Bray-Curtis Anosim.** Analysis of similarities (Anosim) is a non-parametric testing method based on permutation and rank sum tests: Using the adosim function in the vegan package for adosim analysis with permutations=999 based on Bray Curtis Distance.
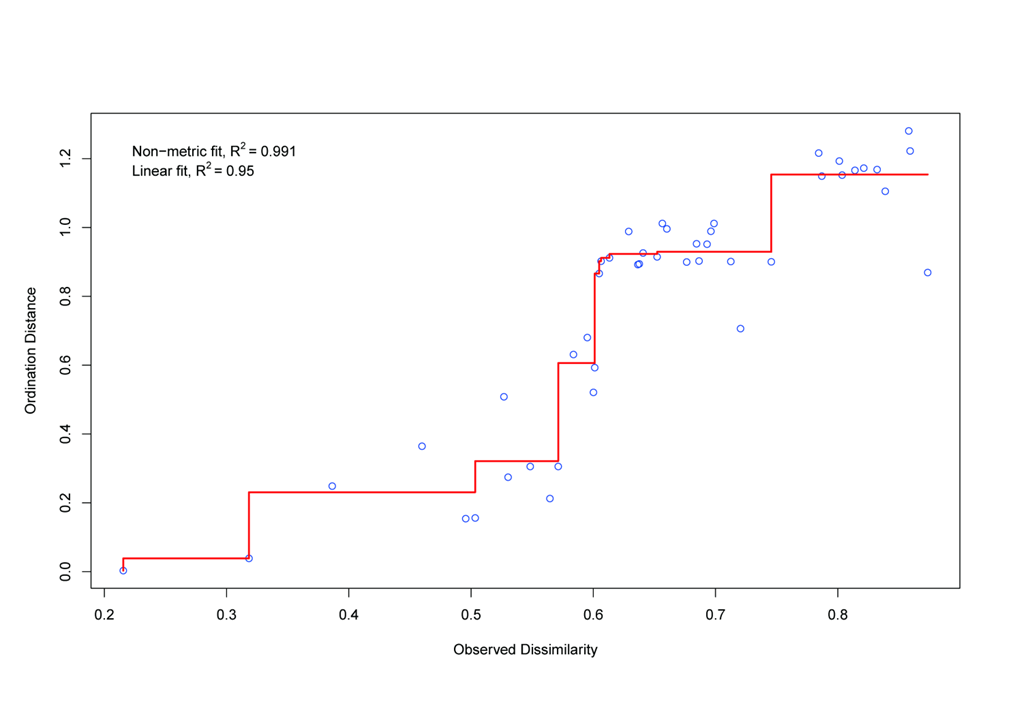


**Supplementary Figure S4. Shepard plot of dimensional reduction results for samples data.** Basically, all points are concentrated near the line segment, indicating a small discrepancy between the reduced distances and actual distances, the dimensional reduction results exhibit high accuracy.
